# Effects of different KRAS mutants and Ki67 expression on diagnosis and prognosis in lung adenocarcinoma

**DOI:** 10.1038/s41598-023-48307-x

**Published:** 2024-02-19

**Authors:** Jun Wang, Liwen Dong, Zhaowei Zheng, Zhen Zhu, Baisheng Xie, Yue Xie, Xiongwei Li, Bing Chen, Pan Li

**Affiliations:** Department of Thoracic Surgery, Hangzhou TCM Hospital Affiliated to Zhejiang Chinese Medical University, Hangzhou, 310007 China

**Keywords:** Non-small-cell lung cancer, Data mining, Data processing, Genome informatics

## Abstract

Lung adenocarcinoma (LUAD) is a prevalent form of non-small cell lung cancer with a rising incidence in recent years. Understanding the mutation characteristics of LUAD is crucial for effective treatment and prediction of this disease. Among the various mutations observed in LUAD, KRAS mutations are particularly common. Different subtypes of KRAS mutations can activate the Ras signaling pathway to varying degrees, potentially influencing the pathogenesis and prognosis of LUAD. This study aims to investigate the relationship between different KRAS mutation subtypes and the pathogenesis and prognosis of LUAD. A total of 63 clinical samples of LUAD were collected for this study. The samples were analyzed using targeted gene sequencing panels to obtain sequencing data. To complement the dataset, additional clinical and sequencing data were obtained from TCGA and MSK. The analysis revealed significantly higher Ki67 immunohistochemical scores in patients with missense mutations compared to controls. Moreover, the expression level of KRAS was found to be significantly correlated with Ki67 expression. Enrichment analysis indicated that KRAS missense mutations activated the SWEET_LUNG_CANCER_KRAS_DN and CREIGHTON_ENDOCRINE_THERAPY_RESISTANCE_2 pathways. Additionally, patients with KRAS missense mutations and high Ki67 IHC scores exhibited significantly higher tumor mutational burden levels compared to other groups, which suggests they are more likely to be responsive to ICIs. Based on the data from MSK and TCGA, it was observed that patients with KRAS missense mutations had shorter survival compared to controls, and Ki67 expression level could more accurately predict patient prognosis. In conclusion, when utilizing KRAS mutations as biomarkers for the treatment and prediction of LUAD, it is important to consider the specific KRAS mutant subtypes and Ki67 expression levels. These findings contribute to a better understanding of LUAD and have implications for personalized therapeutic approaches in the management of this disease.

## Introduction

The prognosis of Lung adenocarcinoma (LUAD) has greatly improved with advancements in molecular subtyping and corresponding treatment methods^1–3^. However, despite advancements, LUAD continues to be a leading cause of cancer-related mortality globally^4–8^, primarily attributable to its high incidence of recurrence and metastasis^9,10^. The Ki67 index in clinical immunohistochemistry is a measure of the growth and proliferation rate of tumor cells. In the context of tumor biology, it is widely acknowledged that a positive correlation exists between the Ki67 index and tumor growth and proliferation rates. Specifically, tumors with a high Ki67 index are typically associated with a high degree of malignancy, which translates into greater aggressiveness and a higher likelihood of distant metastasis. This relationship has been recognized by the World Health Organization (WHO), which in its 5th edition classification, proposed the term “carcinoid tumors with elevated mitotic counts and/or Ki67 proliferation rates” to describe tumors with high Ki67 indices^11^. Numerous studies have recommended the utilization of Ki67 immunohistochemistry for diagnostic testing of bronchial or lung biopsies^12^ making Ki67 a critical indicator for tumor diagnosis and pathological grading. In clinical practice, patients with a high Ki67 index are usually associated with a poor prognosis^13,14^.

Mutations in various genes have been found to be associated with the initiation, development, and metastasis of diverse types of tumors^15,16^. Among the identified mutations, the RAS family of genes is the most common, with KRAS being the most representative^17^. An activating KRAS mutation was discovered in human lung cancer samples in 1984, which were absent in the corresponding normal tissue. Several studies on human cell lines have confirmed this finding. Furthermore, it has been observed that somatic KRAS mutations are frequently present in lung cancers^18^. KRAS encodes a small GTPase membrane-bound protein that exists in two distinct forms: a GDP-bound form in the inactive state and a GTP-bound form in the activated state. Normally, when KRAS binds to GTP, the last phosphate group of GTP is cleaved, converting it to GDP, which inactivates it. However, when the KRAS gene is mutated, the KRAS protein remains activated by binding to GTP, and it no longer depends on the stimulation of superior signals. This results in hyperactive downstream signaling pathways that initiate and maintain hyperactive signaling in tumor genesis^19–21^. It is worth noting that KRAS-activating mutations are not universally carcinogenic in the absence of other interference. Different KRAS mutations may lead to different activation degrees of the Ras signaling pathway, which may be correlative with pathogenesis and prognosis^22,23^. However, the relationship between different subtypes of KRAS mutations and the pathogenesis and prognosis of LUAD remains unclear.

The advent of immune checkpoint inhibitors (ICIs) has revolutionized tumor therapy. Tumor cells have the ability to evade the immune system by activating immune checkpoint pathways that suppress anti-tumor immune responses. The principle behind the use of ICI is to block the co-inhibitory signaling pathway, thereby promoting the immune system to clear tumor cells^24^. Currently, there are no effective targeted therapies for KRAS mutations^25^. Fortunately, studies have shown that patients with KRAS mutations could benefit from ICI^26,27^. Therefore, KRAS mutation status may represent a potential biomarker of clinical benefit from ICI in LUAD^28^. However, it is worth noting that the heterogeneity of KRAS mutation may be a key consideration in the treatment efficacy of ICI in LUAD^29^. A common molecular lesion is a somatic missense KRAS mutation that introduces amino acid substitutions at positions 12, 13, and 61^30^. The mutation typically occurs in exon 2 at codon 12, and is rarely found at exon 3 codon 61^21,31^. Although the development of ICI has changed the treatment prospects of LUAD, but the therapeutic effects are not ideal^32^. Given that different KRAS mutations vary in both form and function, identifying specific KRAS mutations that are responsive to ICIs is a crucial aim of ongoing research.

The tumor mutation burden (TMB) refers to the overall count of detected somatic gene coding errors, base substitutions, gene insertions, or deletions per one million bases^33^. Tumor cells with a high frequency of gene mutations often display an abundance of tumor antigens on their surfaces, making them more susceptible to attacks by the body's immune system. As a result, the tumor mutation burden (TMB) has emerged as a promising biomarker for predicting the effectiveness of immune checkpoint inhibitor (ICI) therapies. Previous research has provided evidence that TMB can serve as a predictive factor for the clinical response to ICI treatments^34^. In addition, we also conducted an analysis to investigate the relationship between the Ki67 immunohistochemical index and TMB.

In this study, we aim to address the critical need for accurate identification and characterization of missense mutations in the KRAS gene in LUAD. While previous research has established the significance of KRAS mutations in lung cancer development and progression, there remains a lack of clarity regarding the specific types and consequences of these mutations. Our study seeks to fill this knowledge gap by comprehensively analyzing missense mutations in the KRAS gene using two targeted next-generation sequencing (NGS) panels. By doing so, we aim to provide a comprehensive understanding of the landscape of missense mutations in KRAS and their potential implications in lung cancer. This study's novelty lies in its characterization of mutations, shedding light on their prevalence, distribution, and potential functional consequences. Ultimately, our findings have the potential to contribute to the development of targeted therapies and personalized treatment approaches for LUAD patients with KRAS missense mutations.

## Materials and methods

### Data preparation

This study included a cohort of 63 patients who underwent immunohistochemistry (IHC) at Hangzhou Hospital of Traditional Chinese Medicine between November 2020 and January 2022. The mutational status of 9 genes or 1059 genes was determined using next-generation sequencing (NGS). Specifically, two panels were utilized for NGS testing: the 9-gene panel and the 1059-gene panel. The latter includes the genes in the former, and detailed gene information for both panels can be found in Supplementary Table 1. For the purposes of this study, our focus was on the 9 genes that were included in both panels.

The study adhered to the ethical principles outlined in the Declaration of Helsinki (revised in 2013). Approval for this retrospective study was obtained from the medical ethics committee of Hangzhou TCM Hospital (Approval No. 2023KLL050). Given the retrospective design of the study, informed consent was not required by the medical ethics committee of Hangzhou TCM Hospital.

DNA was extracted from the case samples obtained. DNA was extracted from the tumor and the blood. The Rapid DNA FFPE Kit (ZYMO) was used for extraction from formalin-fixed paraffin-embedded (FFPE) sections. Qubit dsDNA HS Analysis Kit (LIFE) and agarose gel electrophoresis were used for DNA quantification. DNA library construction was completed using the ABclonal lone Rapid DNA Library Preparation Kit according to the manufacturer’s protocol. Covaris S220 was used to physically cut 50-200 ng DNA for A-tailing, connector ligation, and PCR amplification. The mixture was hybridized with Boke’s hybridization probe for 16 to 18 h at 65 °C, followed by capture using M270 strand avidin beads for 45 min. Then 15 cycles of post-capture amplification were performed to obtain the capture library. All samples were sequenced using two NGS testing panels that included 1059 cancer-related genes. The panel used for each sample is listed in Supplementary Table 2. Trimmomatic is used to filter low-quality reads and BWA MEM is used to map reads. GATK SortSam, MarkDuplicates, CollectHsMetrics, BaseRecalibrator, ApplyBQSR, AnalyzeCovariates, CollectSequencingArtifactMetrics, Mutect2, GetPileupSummaries, CalculateContamination, FilterMutectCalls, FilterByOrientationBias is used for mutation calling and filtering.

The transcriptomic data from LUAD patients were obtained from The Cancer Genome Atlas (TCGA) database. Specifically, RNA-seq data was available for 496 patients with LUAD from the TCGA portal (https://portal.gdc.cancer.gov/). In addition, MSK data of NSCLC patients on TMB results and survival data were directly downloaded from the cBioPortal for Cancer Genomics (https://www.cbioportal.org/study/summary?id=msk_impact_2017). These data sources were chosen for their comprehensive and reliable collection of genomic and clinical data of patients with lung cancer, which enabled us to conduct a thorough analysis of the biomarkers of interest in our study.

### Local clinical cohorts

Clinical patient information included gender, age, smoking history, pathological stage, and immunohistochemical scores of Ki67, NapsinA, TTF, EGFR, and P53. Detailed clinical data can be found in Supplementary Table 2. Pathological staging was evaluated by experienced pathologists using the TNM staging system. Staging and pathological diagnosis were manually organized based on pathological reports. The ggplot2 package in R is used to draw bar charts and violin charts.

### Immunohistochemistry (IHC) analysis of biomarkers

IHC was performed to analyze the expression of specific biomarkers TTF, p53, EGFR, NapsinA, and Ki67 in lung cancer tissue samples. Briefly, tissue sections were deparaffinized, rehydrated, and subjected to antigen retrieval. Endogenous peroxidase activity was blocked with hydrogen peroxide, and non-specific binding was blocked with serum. The tissue sections were then incubated with primary antibodies against the biomarkers of interest, followed by secondary antibodies conjugated with horseradish peroxidase. The IHC staining was visualized with diaminobenzidine (DAB) and counterstained with hematoxylin. Images were captured using a microscope equipped with a digital camera. The binary variables of conventional IHC biomarkers used for statistical analysis were as follows: positive or negative for TTF, p53, EGFR, and NapsinA; proliferation index of Ki67. The Ki67 index was determined by counting at least 200 cells within the area of highest Ki67 expression.

### Division of missense and non-missense mutations

We partitioned the samples into two distinct cohorts, distinguishing between those harboring KRAS missense mutations and those with non-missense mutations, with the aim of ascertaining the utility of KRAS missense mutations as potential biomarkers for ICI therapy. A missense mutation is characterized by the alteration of a given codon, such that it results in the substitution of the original codon with a novel one, encoding a different amino acid and consequently leading to a modification in the amino acid sequence. In this study, we considered all mutations that bring about changes in amino acids, ascertained through the Genome Analysis Toolkit (GATK) analysis, as falling under the category of missense mutations. It is noteworthy that KRAS mutations predominantly manifest as single-base missense mutations, with a significant proportion clustering within three well-defined hotspots located at codon 12, 13, and 61^35,36^. In the NGS panels, probes are designed specifically targeting these KRAS missense mutation sites. In both the TCGA and MSK datasets, the original missense mutation labels obtained from the database are utilized. Non-missense mutations, on the other hand, pertain to the samples that fall outside the category of missense mutations.

### mRNA expression profile and enrichment analysis

Correlation analysis of gene expression was conducted using the ggplot2 package in R. Differential gene analyses were performed using the DESeq2 package to associate gene signatures with KRAS missense or the control group. Gene sets from the MsigDB database were obtained using the msigdbr package in R. Enrichment functional pathway maps were generated using the ClusterProfiler package. Heat maps were created using the pheatmap package.

### TMB and survival analysis

The local clinical data TMB was defined as the ratio of the number of somatic nonsynonymous variants in the coding regions, including indels and deletions, detected in tumor tissue to the size of the region that was analyzed. TMB results from the TCGA data were calculated using the maftools package. TMB results and survival data from the MSK database were downloaded directly. The IHC score of Ki67 and the expression of Ki67 were classified based on the median value. Survival analysis and visualization were performed using the Survminer package in R.

### Statistical analysis

Data analysis was conducted using R version 3.6.0. The Wilcoxon test was employed to compare continuous variables, including gene expression values, IHC Ki67 scores, and TMB. Kaplan-Meier survival curves were generated and the log-rank test was performed using the Survminer package in R. Significance tests in this study were two-sided, with a p-value <0.05 indicating statistical significance. A p-value <0.01 was deemed marginally significant.

## Result

### Clinical data showed that age and pathological stage were correlated with the IHC Ki67 score

**Figure 1:**
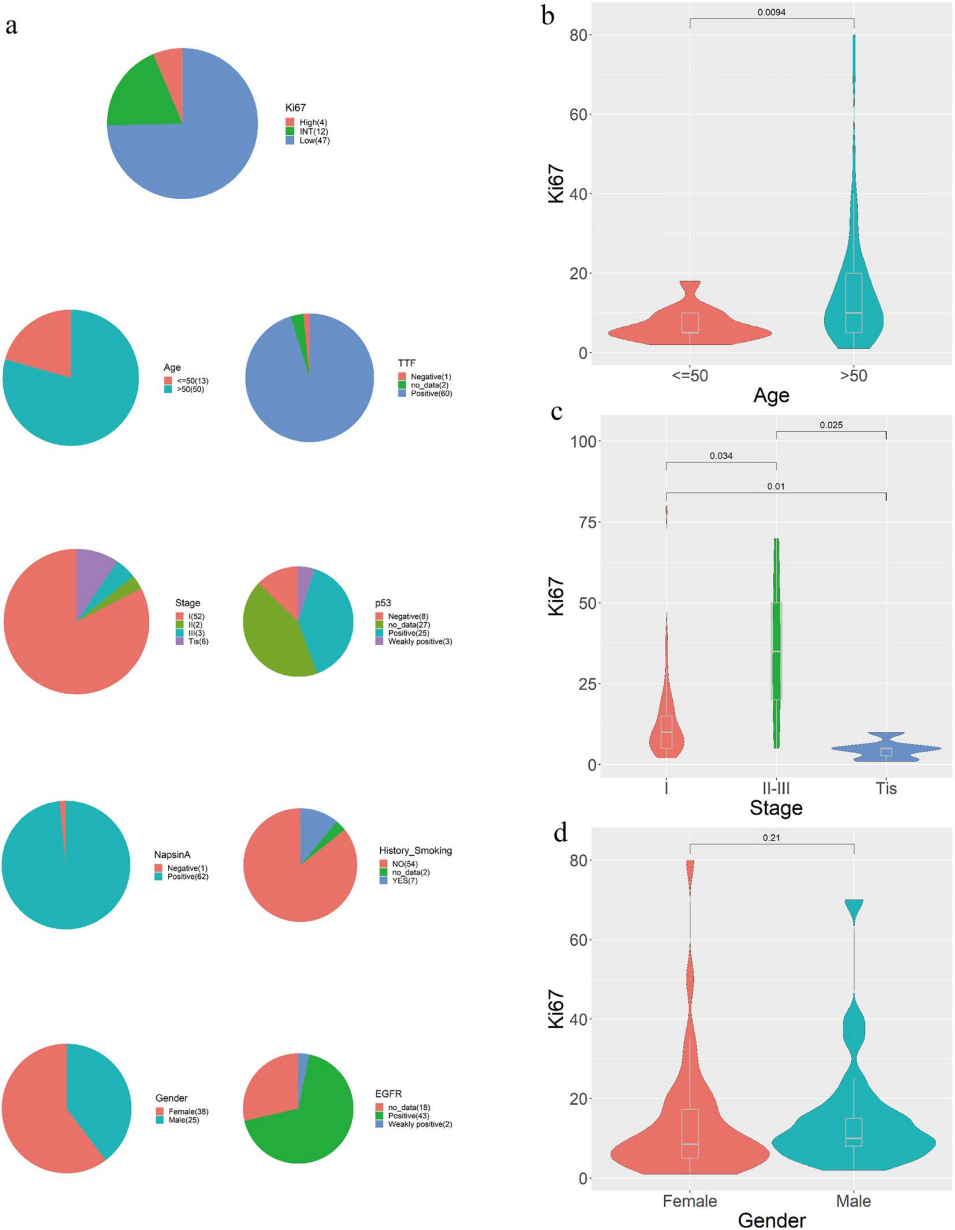
Clinical information of patients with lung adenocarcinoma. (**a**) Clinical information and immunohistochemical results of patients with lung adenocarcinoma. (**b**–**d**) Ki67 immunohistochemical distribution of lung adenocarcinoma patients with different ages, pathological stages, and sex.

Ki67 is a proliferating cell-associated antigen whose function is closely related to mitosis. It is indispensable in cell proliferation, so the Ki67 proliferation index is an important indicator of immunohistochemistry in pathology. We collected clinical information from 63 NGS samples (including 38 9-gene samples and 25 1059-gene samples) and analyzed these samples. Clinical information included gender, age, smoking history, pathological stage, and immunohistochemical results of the samples (Fig. 1a). The results of clinical information analysis showed that the Ki67 immunohistochemical index of people over 50 years old was significantly higher than that of people under 50 years old (P = 0.0094, Wilcox test) (Fig. 1b). Patients with Tis stage were significantly lower than patients with stage I (P = 0.01, Wilcox test) and patients with stage II-III (P = 0.025, Wilcox test), and patients with stage I were significantly lower than patients with stage II-III (P=0.034, Wilcox test)) (Fig. 1c). There was no significant difference between the sexes in the Ki67 index (P=0.21, Wilcox test) (Fig. 1d).

### There were significant differences in Ki67 scores between KRAS missense and non-missense mutations

**Figure 2:**
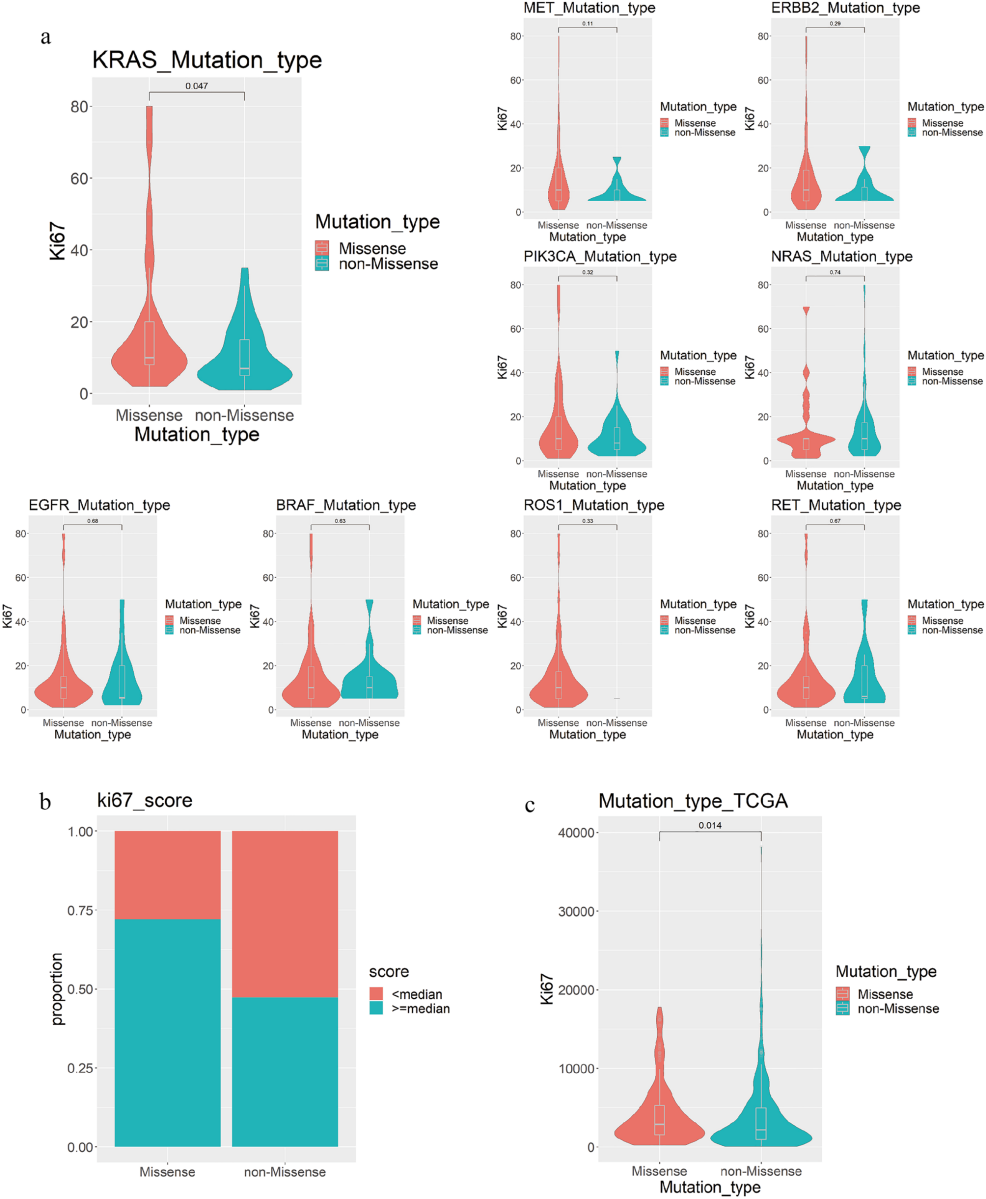
The relationship between different gene mutation types and Ki67 IHC score. (**a**) Distribution of Ki67 IHC scores for different mutation types of each gene. (**b**) Proportion of clinical patients with different KRAS mutation types in the Ki67 IHC score interval. (**c**) Distribution of Ki67 expression corresponding to different KRAS mutation types in TCGA data.

At the same time, 63 clinical patient samples with 9 shared gene tests and Ki67 IHC indicators were analyzed, and the relationship between samples with different gene mutation types and Ki67 IHC index was analyzed. The results showed that the Ki67 immunohistochemical index of the KRAS gene missense mutation samples was significantly higher than that of the KRAS non-missense mutation samples index (P = 0.047, Wilcox test), while other genes were not statistically different (Fig. 2a). Among the 63 patients with LUAD with Ki67 immunohistochemical index, the proportion of patients with Ki67 immunohistochemical index (≥ median) in the KRAS gene missense mutation group was higher than that in the non-missense mutation group (18/25 vs 18/38) (Fig. 2b). In the TCGA data, the expression level of Ki67 (MKi67) in KRAS gene missense mutation samples was significantly higher than that in KRAS gene non-missense mutation samples (P=0.014, Wilcoxon test) (Fig. 2c).

### TCGA data correlation analysis showed that KRAS expression was significantly positively correlated with Ki67 expression. Enrichment analysis showed that both KRAS and Ki67 activated SWEET LUNG CANCER KRAS DN and CREIGHTON ENDOCRINE THERAPY RESISTANCE 2 pathways

**Figure 3:**
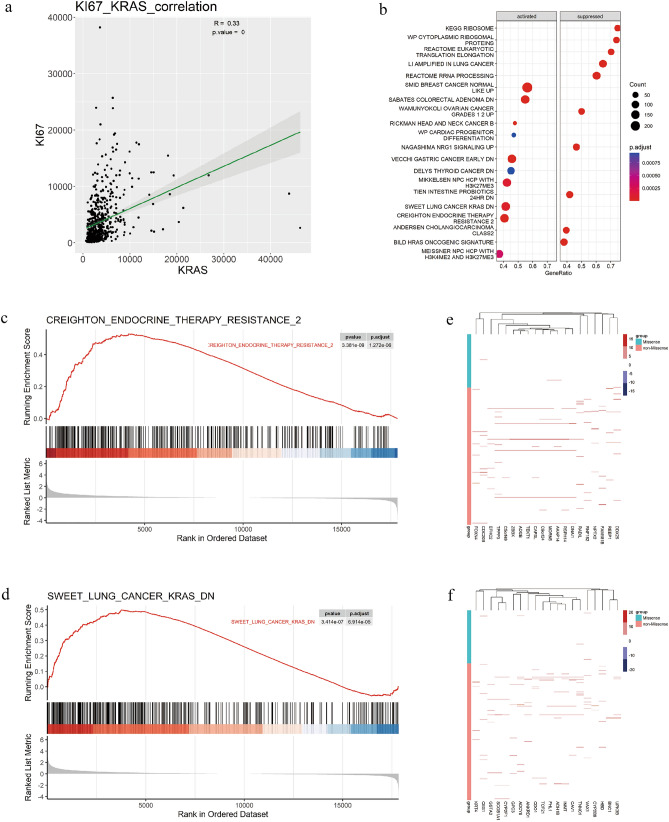
GSEA analysis of mRNA relative expression levels of signal pathways enriched in TCGA dataset. (**a**) Correlation analysis results of KRAS gene expression and Ki67 gene expression in TCGA dataset. (**b**) Enrichment analysis showed that SWEET LUNG CANCER KRAS DN and CREIGHTON ENDOCRINE THERAPY RESISTANCE 2 pathways were activated. (**c**,**d**) enrichment plot regarding two pathways. (**e**,**f**)Heat map of relative mRNA expression of the two pathways.

We performed a correlation analysis on the expression of KRAS and Ki67 (MKi67) in the TCGA data, and the results showed that they had a statistically significant positive correlation at the RNA level (r = 0.33, P < 0.0001, Spearman) (Fig. 3a). To further explore the difference between KRAS missense and non-missense mutation subtypes, we performed GSEA based on RNA-seq data from TCGA, which revealed a prominent enrichment in KRAS-missense-mutant group of signatures related to SWEET LUNG CANCER KRAS DN (P < 0.0001) and CREIGHTON ENDOCRINE THERAPY RESISTANCE 2 (P< 0.0001) (Fig. 3b–d). We generated heat maps to depict the distribution of RNA levels in the corresponding pathway between the KRAS missense-mutant and non-missense-mutant groups (Fig. 3e,f).

### KRAS missense mutation and Ki67 expression were significantly increased at the TMB level compared with the other groups

**Figure 4:**
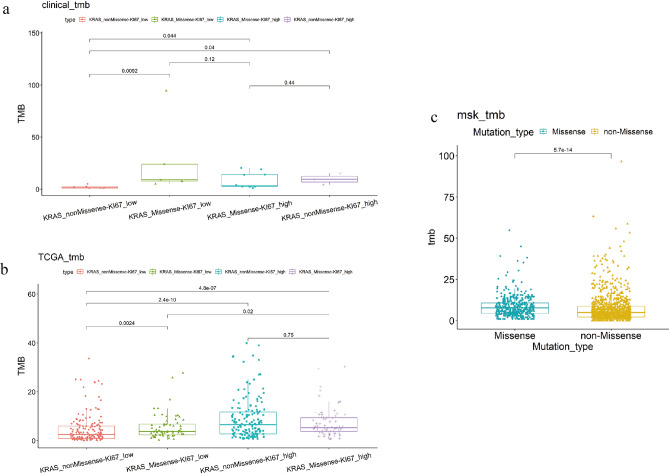
Distribution of TMB results in different data sets with different KRAS mutation types. (**a**) TMB distribution of different KRAS mutation types in the clinical dataset. (**b**) TMB distribution of different KRAS mutation types in the TCGA dataset. (**c**) TMB distribution of different KRAS mutation types in the MSK dataset.

Samples with different KRAS mutations and Ki67 expression in clinical data, TCGA data, and MSK data were genotyped. Clinical data showed that the KRAS non-missense mutation and Ki67 IHC low score group were significantly lower than the other groups (P < 0.05, Wilcox test) (Fig. 4a). Also, TCGA data showed that KRAS non-missense mutation and Ki67 IHC low score group were significantly lower than other groups (P < 0.05, Wilcox test). The results also showed that KRAS missense mutation and Ki67 low IHC score group were significantly lower than KRAS missense mutation and Ki67 high IHC score group (P = 0.02, Wilcox test) (Fig. 4b). In MSK data, the TMB of KRAS missense mutation samples was significantly higher than that of KRAS non-missense mutation samples (P < 0.0001, Wilcox test) (Fig. 4c).

### Survival analysis showed that the survival time of KRAS missense mutant samples was longer than that of non-missense mutant samples, and the survival time of Ki67 high-expression samples was longer than that of low-expression samples

**Figure 5:**
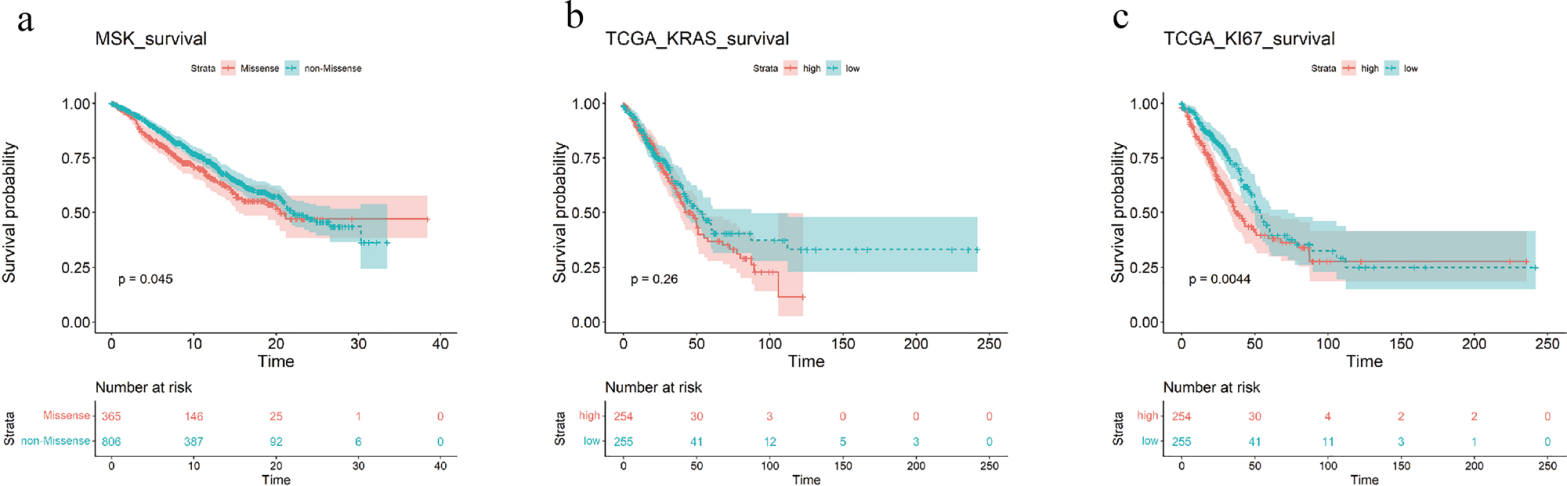
Survival analysis results of MSK and TCGA data. (**a**) Survival analysis of different KRAS mutation types in the MSK dataset. (**b**) Survival analysis of different KRAS mutation types in TCGA data sets. (**c**) Survival analysis of different Ki67 expressions in TCGA data sets.

Survival analysis can study the influence of different traits on the survival time of patients. Survival analysis is performed on MSK and TCGA data. MSK data showed that the survival time of KRAS missense mutant samples was significantly longer than that of non-missense mutant samples (P = 0.045, Wilcox test) (Fig. 5a). TCGA data showed that the survival time of KRAS missense mutant samples was longer than that of non-missense mutant samples (Fig. 5b), and the survival time of Ki67 high-expression samples was longer than that of low-expression samples (P = 0.0044, Wilcox test) (Fig. 5c).

## Discussion

The Ki67 marker, which is related to tumor cell proliferation, has been found to be associated with the progression, metastasis, and prognosis of LUAD^37^. In our analysis of clinical patient information, it was observed that age and pathological stage were significantly correlated with the Ki67 IHC index, which is consistent with previous studies. However, gender was not found to be correlated with the Ki67 IHC index, possibly because the pathogenesis of LUAD is not solely related to smoking. Further research is necessary to explore this issue by collecting more samples and life information.

The utilization of ICIs have revolutionized cancer treatment by enhancing the immune system to target tumor cells^38^. The function of ICI is to disrupt the inhibitory signals between tumor cells and immune effector cells, allowing activated T cells to target these tumor cells^39^. Previous studies have shown that gene mutations in KRAS, FGFR, RET, BRAF, and HER2 have an impact on clinical benefits of ICI in advanced non-small cell lung cancer^40^. Although previous studies have found that KRAS mutations impact the treatment of ICI, they did not consider the heterogeneity of KRAS^41^. Therefore, various theories exist regarding the evaluation of KRAS mutations as ICI biomarkers. In this study, the samples were divided into KRAS missense and non-missense mutations. To ensure comprehensive analysis, data sets from three different sources were used. The Ki67 IHC index, an important indicator of immunohistochemistry, also showed a difference between KRAS missense and non-missense mutations in the study.

Among the 63 cases of LUAD patients, a significant difference in the Ki67 immunohistochemical index was observed only between samples with KRAS gene missense mutations and non-missense mutations. Notably, a higher proportion of patients in the missense mutation group exhibited a Ki67 immunohistochemical index equal to or greater than the median value. These findings underscore the distinctive nature of KRAS mutations compared to other mutation types, as well as variations within different forms of KRAS mutations. It is important to note that our study focused solely on investigating differences within the mutant forms of KRAS itself and did not consider the potential interactions between KRAS and other mutations. Further studies are warranted to explore these interactions in greater detail.

Additionally, we conducted an analysis using the TCGA dataset. The results revealed that the expression of Ki67 in samples with KRAS gene missense mutations was significantly higher compared to samples with non-missense mutations. This finding supports the notion that different expression types of the KRAS gene exhibit distinct characteristics, aligning with previous research. Notably, there was a significant positive correlation between KRAS gene expression and Ki67 gene expression. This suggests that elevated expression of both genes may be associated with the pathogenesis of lung adenocarcinoma. Furthermore, enrichment analysis highlighted the co-activation of the SWEET LUNG CANCER KRAS DN pathway by KRAS and Ki67, providing further confirmation of our observations. In the future, it might interesting to predict drugs can target both KRAS mutation and Ki67 using some machine leanring^42–44^ and experimental methods^45^.

Survival analysis was conducted using the MSK and TCGA data.The results revealed that KRAS missense mutant samples had longer survival times than the control samples, while samples with high expression of Ki67 had longer survival times compared to those with low expression.This suggests that Ki67 can serve as an indicator of KRAS activation, thus enhancing the accuracy of survival time prediction.

Furthermore, the oncogenic KRAS gene mutation not only directly facilitates the proliferation and survival of tumor cells but also exerts an impact on the tumor microenvironment. It has been observed by researchers that the combination of a KRAS G12C inhibitor and an immune checkpoint inhibitor (ICI) can effectively suppress certain types of immunogenic lung cancer^46^. The efficacy of the combination therapy might be related to the TMB. TMB is indicative of the overall number of mutations present in tumor cells. A higher frequency of gene mutations in tumor cells results in an increased expression of tumor antigens on the cell surface. Consequently, tumor cells become more susceptible to immune system-mediated attack, thereby enhancing the effectiveness of immune checkpoint inhibitors (ICI)^47^. Different KRAS mutations may have different effects on TMB. We compared the TMB values of clinical data, TCGA data, and MSK data for samples with different KRAS genotypes. The high expression and low expression of Ki67 obtained by KRAS missense mutation and non-missense mutation and median differentiation resulted in four groups of TMB: Kras_missense-ki67_high, KRAS_MISsense-Ki67_low, KRAS_NONMISSENse-Ki67_high, KRAS_NONMISsense-Ki67_low. The results showed that TMB in the KRAS_nonmissense-Ki67_LOW group was significantly lower than that in the other three groups, and there was no statistical difference between the other three groups. The TCGA data were consistent with the clinical data, revealing that the TMB in the KRAS_MISsense-Ki67_low group was significantly lower compared to the KRAS_MISsense-Ki67_HIGH group. In the MSK data, the TMB of samples with KRAS missense mutations was significantly higher than that of samples with KRAS non-missense mutations. In all three datasets, it was consistently observed that the TMB of samples with KRAS missense mutations was significantly higher than those without missense mutations. Additionally, the TMB was found to be higher in samples with high Ki67 expression compared to those with low Ki67 expression. These findings indicate that KRAS gene missense mutations and high Ki67 expression contribute to an increased TMB level, which can potentially serve as a predictive marker for the efficacy of immunotherapy.

Our study demonstrates a significant correlation between KRAS mutant subtypes, Ki67 expression, and increased TMB levels. However, further investigations are necessary to elucidate the underlying mechanisms.Secondly, our study utilized bulk sequencing, disregarding cell heterogeneity^48,49^. Conducting a single-cell resolution study would be more appropriate for a comprehensive investigation of the mechanisms underlying KRAS mutation, Ki67, and TMB.Additionally, the presence of differences among various databases is inevitable. Nonetheless, the consistent results across different databases lend credibility to our findings. Furthermore, the clinical dataset we used included a small sample size, rendering the data statistically insignificant. Hence, additional data is required for further investigation.

## Conclusion

In conclusion, our study revealed significant variations in immunohistochemical Ki67 scores among different KRAS variants. Furthermore, we observed a positive correlation between KRAS expression and immunohistochemical Ki67 scores. Interestingly, Ki67 expression proved to be a more effective prognostic indicator than KRAS mutation status. Additionally, our data demonstrated a link between KRAS missense mutations, higher Ki67 scores, and increased TMB. This finding suggests a potential impact on the efficacy of subsequent immunotherapy. Moreover, integrating Ki67 scores and KRAS mutation type improved the accuracy of survival prediction.

### Supplementary Information

Supplementary Table 1.

Supplementary Table 2.

## Data Availability

TGCA and MSK data are publicly avaiable from https://portal.gdc.cancer.gov/ and https://www.cbioportal.org/study/summary?id=msk_impact_2017. The variant data for this study have been deposited in the Genome Variation Map (GVM) in National Genomics Data Center, Beijing Institute of Genomics, Chinese Academy of Sciences and China National Center for Bioinformation, under accession number GVM000561. (https://bigd.big.ac.cn/gvm/getProjectDetail?Project=GVM000561). The data generated during the current study available from the corresponding author on reasonable request.
